# Severe acute respiratory syndrome coronavirus‐2 (SARS‐CoV‐2) infection after allogeneic stem cell transplantation

**DOI:** 10.1002/ccr3.2984

**Published:** 2020-06-10

**Authors:** Takashi Onaka, Fumie Iwai, Aiko Kato‐Ogura, Akihito Yonezawa

**Affiliations:** ^1^ Department of Hematology Kokura Memorial Hospital Fukuoka Japan

**Keywords:** allogeneic transplantation, COVID‐19, CT findings, immunocompromised host, malignant lymphoma

## Abstract

This is the first report of a case of COVID‐19 after allogeneic stem cell transplantation. Our case suggests that COVID‐19 may exist without characteristic CT images, especially in immunocompromised hosts, such as patients after transplantation.

## Graphical abstract

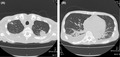

## 1 INTRODUCTION

Severe acute respiratory syndrome coronavirus‐2 (SARS‐CoV‐2) is a novel coronavirus first detected in Wuhan, China. The virus causes coronavirus disease 2019 (COVID‐19). Over 1 000 000 cases of COVID‐19 have been confirmed worldwide.^1^ Here, we report the first case of COVID‐19 after allogeneic stem cell transplantation.

## 2 CASE EXAMINATION

**Figure 1:**
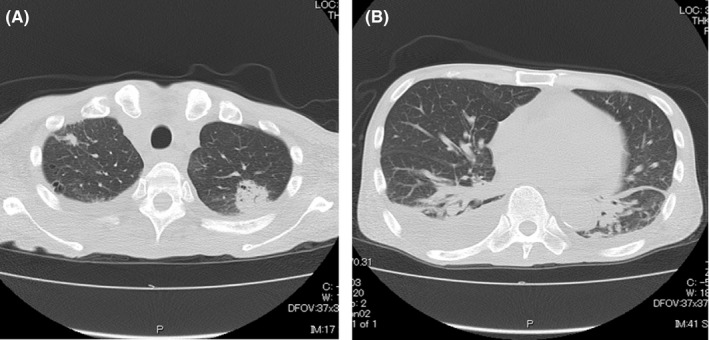
A, Chest CT findings. Organizing pneumonia was observed in both upper lobes. B, Chest CT findings. Bilateral pleural effusion was present

A 61‐year‐old male with diffuse large B‐cell lymphoma transformed from follicular lymphoma underwent peripheral blood stem cell transplantation (PBSCT) from his HLA haploidentical daughter. He underwent de‐escalation of immunosuppressant drugs because of early relapse after PBSCT. The duration of chronic graft‐vs‐host disease (GVHD) was extended, but he did not need additional therapy. At day 205 after PBSCT, he had a fever of 100°F and a wet cough. He visited our hospital because his symptoms persisted for 2 days. He had not travelled to a foreign country nor had contact with anyone with COVID‐19. His chest X‐ray showed no apparent bacterial pneumonia, and a CT scan showed only small nodules that were diagnosed as scar tissue from past organizing pneumonia and pleural effusion (Figure 1). Although he was radiographically atypical for COVID‐19, a COVID‐19 PCR test was performed on a nasopharyngeal swab. Laboratory tests showed leucopenia (WBC 1000/µL), neutropenia (ANC 20/µL), a high procalcitonin level (8.94 ng/mL), and a high CRP level (26.3 mg/dL). He was hospitalized and started taking antibiotics with a diagnosis of community‐acquired pneumonia. PCR was positive the day after hospitalization. By the ninth day in the hospital, his respiratory condition had not worsened.

## 3 DISCUSSION

Currently, COVID‐19 is spreading around the world, and typical COVID‐19 clinical features and imaging patterns on chest CT have been reported. Although common symptoms of COVID‐19 include fever and respiratory symptoms, a report indicated that COVID‐19 was diagnosed without showing the typical symptoms.^2^ They reported a patient was admitted to their hospital because of a temporary loss of consciousness, and no respiratory symptoms or fever was observed. Furthermore, their report suggested that COVID‐19 diagnosis by symptoms alone can be difficult. On the other hand, in the image findings, Chinese researchers revealed bilateral lung opacities on chest CT in COVID‐19‐infected patients and described lobular and subsegmental areas of consolidation as the most typical findings.^3^ Another study found that the hallmarks of COVID‐19 on imaging were bilateral and peripheral ground‐glass and consolidative pulmonary opacities.^4^ However, in our patient, we did not find typical findings on CT. This is the first report of a case of COVID‐19 after allogeneic stem cell transplantation. The absence of the characteristic imaging features might be related to leucopenia or immunosuppression. Our case suggests that COVID‐19 may exist without characteristic CT images, especially in immunocompromised hosts, such as patients after transplantation.

## 4 CONCLUSION

Although there are many unclear points about COVID‐19, typical features may not be present on images, and it is important to note the possibility of infection, especially in immunocompromised patients.

## CONFLICT OF INTEREST

None declared.

## AUTHOR CONTRIBUTIONS

TO: managed the patient and wrote the manuscript. FI, AO, and AY: co‐ordinated and approved the final version of the manuscript.
